# Skeletal Muscle Myofibers Directly Contribute to LPS-Induced Systemic Inflammatory Tone

**DOI:** 10.3389/fphar.2022.917917

**Published:** 2022-06-23

**Authors:** Joseph J. Bivona III, Madeleine M. Mank, Renee D. Stapleton, D. Clark Files, Michael J. Toth, Matthew E. Poynter

**Affiliations:** ^1^ Department of Medicine and Vermont Lung Center, University of Vermont Larner College of Medicine, Burlington, VT, United States; ^2^ Department of Internal Medicine, Section on Pulmonary, Critical Care, Allergy and Immunology, Wake Forest School of Medicine, Winston-Salem, NC, United States

**Keywords:** skeletal muscle, cytokine, endotoxin, TLR4**–toll-like receptor 4**, LPS (lipopolysaccharide), inflammation, sepsis

## Abstract

The abundance, anatomical distribution, and vascularity of skeletal muscle make it a potentially important contributor to local cytokine production and systemic cytokine abundance during inflammatory events. An orchestrated balance between the production of pro- and anti-inflammatory mediators is necessary for proper immune function, yet the contribution of the body’s largest organ system, comprised primarily of skeletal muscle myocytes that fuse to form myofibers, to this process is largely unknown. Endotoxin (lipopolysaccharide, LPS) stimulates toll-like receptor 4 (TLR4) to induce the production of several pro-inflammatory cytokines, including interleukin-6 (IL-6) and C-C motif chemokine ligand 2 (CCL2), by a of myriad cell types. We sought to quantify the influence of myofibers on systemic cytokine concentrations following an acute endotoxemia challenge. To accomplish this, we generated muscle specific conditional knockouts for TLR4 (TLR4SMKO), IL-6 (IL6SMKO), and CCL2 (CCL2SMKO). We administered low concentrations of intravenous LPS (IV LPS) to these receptor and effector knockout mice and collected samples after 3 h. Using gene expression analysis of gastrocnemius muscle and serum cytokine measurements after IV LPS, we determined that deletion of myofiber IL-6 or CCL2 led to a 93% and 57% reduction of these specific cytokines in the systemic circulation, respectively. Myofiber specific TLR4 deletion decreased the expression of IL-6, CCL2, and C-X-C motif chemokine ligand 1 (CXCL1) in the gastrocnemius muscle. These data indicate the critical involvement and direct contribution of myofibers during the early systemic inflammatory cytokine response to endotoxin.

## Introduction

Systemic cytokine balance can influence the outcomes of critically ill patients with sepsis. Whereas a robust and appropriately-timed immune response is required for recovery from critical illness, excess pro- and anti-inflammatory cytokines can impair recovery by injuring tissues or diminishing the immune response, respectively (Chaudhry et al., 2013). Dysregulated cytokine secretion is associated with organ dysfunction in sepsis (Tisoncik et al., 2012). During this response, blood coagulates (Schouten et al., 2008), vasculature permeabilizes (Ince et al., 2016), and overactivated leukocytes induce non-specific organ damage (Czaikoski et al., 2016). Together, these processes result in poor outcomes, including increased morbidity, mortality, and long-term functional impairment in survivors, which are, in part, mediated by elevated concentrations of the cytokines interleukin-1β (IL-1β), IL-6, IL-8 (CXCL8), and macrophage chemoattractant protein-1 (MCP-1, CCL2) (Li et al., 2017; Matsumoto et al., 2018). While a formal definition of cytokine release syndrome or “cytokine storm” is disputed, it can be broadly described phenotypically as the induction of systemic inflammation leading to multiorgan dysfunction (Fajgenbaum and June, 2020). During infection, the generation of cytokines is typically attributed to leukocytes, mostly macrophages and T lymphocytes (Fajgenbaum and June, 2020). However, cells other than leukocytes that constitute the bulk of most solid organs are also contributors to local cytokine production and systemic cytokine concentrations. Crosstalk between immune- and non-immune cells within tissues further blurs this line. With the curation of protein and gene expression databases (Uhlén et al., 2015), there is a renewed appreciation that tissues previously thought to be non-immunomodulating, may possess the ability to produce both pro- and anti-inflammatory cytokines.

Skeletal muscle is a tissue that potently upregulates cytokines, termed “myokines”, after exercise, which contribute to nutrient balance and the repair of damaged myofibers (Pedersen et al., 2007). IL-6 was the first myokine to be discovered and increases 100-fold after an acute exercise bout (Pedersen et al., 2003). Muscle derived IL-6 functions differently following exercise than in inflammatory settings. During an infection, IL-6 activates the JAK/STAT pathway to propagate immune activation and direct adhesion of leukocytes (Chen et al., 2006; Scheller et al., 2011). Following exercise, IL-6 signals to adipocytes to liberate free fatty acids and causes the liver to increase gluconeogenesis. IL-6 also evokes autocrine signals to increase glucose uptake (Laurens et al., 2020).

The effects of exercise can also have a positive impact on lung injury. For example, forced exercise after intratracheal (IT) LPS administration attenuated neutrophilic lung injury (Files et al., 2015). This decrease was found to be due to an exercise induced reduction of granulocyte colony stimulating factor (G-CSF). Furthermore, a reduction of circulating G-CSF was seen in patients with respiratory failure undergoing early mobility therapy compared to controls who received no exercise. Since skeletal muscle tissue potently secretes pro-inflammatory cytokines, composes up to 40% of the body’s mass (Griffin and Goldspink, 1973), and is an important storage organ for amino acids that are liberated during critical illness (Biolo et al., 1995; Wolfe, 2006), understanding the contribution of myofibers to systemic inflammatory tone could provide information for muscle-centric therapeutic strategies for critically ill patients.

Using a model of acute lung injury, our recent work showed that muscle potently expresses and releases several pro-inflammatory cytokines and is synergized by macrophages (Bivona et al., 2021). In this model, bacterial LPS is delivered intratracheally, causing cellular influx to the lung between 6 and 12 h, which persists for several days. Interestingly, skeletal muscle transiently produces cytokines, peaking at 3 h after injury. Mechanistic *in vitro* studies showed that myotubes synergistically increase their cytokine output with macrophages to amplify LPS-induced pro-inflammatory cytokine production through macrophage derived tumor necrosis factor α (TNFα), yet the extent to which muscle feeds into the systemic cascade *in vivo* is incompletely understood (Laitano et al., 2021a; Laitano et al., 2021b).

To test the hypothesis that myofibers are a sensor of and cytokine-producing responder to TLR4-activating stimuli during the initial phase of systemic inflammation, we created a series of myofiber-specific knockout mice and subjected them to an acute endotoxin challenge. Animals containing modified estrogen receptor-Cre fusion (MerCreMer) constructs driven by a human skeletal muscle actin promoter (HSA-MCM) were crossed with mice containing loxP sites surrounding exons in toll-like receptor 4 (*Tlr4*, TLR4SMKO), interleukin-6 (*Il6,* IL6SMKO), and c-c motif chemokine ligand 2 (*Ccl2*, macrophage chemoattractant protein 1, *Mcp1*, CCL2SMKO) genes.

Tamoxifen-induced deletion confirmed selective genomic gene disruption in myofibers. Three hours after IV LPS administration to wild type and gene-disrupted mice, gastrocnemius gene expression of *Tlr4*, *Il6, Ccl2,* and *Cxcl1* were significantly decreased in TLR4SMKO mice. Luminex-based serum cytokine analysis showed significant and substantial decreases in circulating IL-6 and CCL2 concentrations in IL6SMKO and CCL2SMKO mice, respectively. Together, these data add to growing evidence (Laitano et al., 2021a; Laitano et al., 2021b) that myofibers constitute an essential responder to systemic pro-inflammatory agonists and contributor to early systemic inflammatory tone.

## Methods

### Mice

Animals were housed in AAALAC-accredited animal facilities at the University of Vermont, and experimental animal procedures were approved by the University of Vermont Institutional Animal Care and Use Committee (PROTO202000223). Mice were maintained on a 12 hour-light/dark cycle, beginning at 07:00 and 19:00, respectively, and provided irradiated chow (Prolab RMH 3000, Cat # 3005984-712, LabDiet, St. Louis, MO) and autoclaved drinking water *ad libitum*.

Myofiber-specific gene knockout mice were generated by selectively breeding heterozygous Tg (ACTA1-cre/Esr1*)2Kesr/J (Human skeletal actin promoter driven MerCreMer double fusion protein, HSA-MCM, The Jackson Laboratory Cat # 025750, Bar Harbor, ME) with either B6(Cg)-Tlr4tm1.1Karp/J, (TLR4^fl/fl^, The Jackson Laboratory Cat # 024872) or B6. Cg-Ccl2tm1.1Pame/J (CCL2^fl/fl^, The Jackson Laboratory Cat # 016849) or IL-6^fL/fL^ mice (Quintana et al., 2013; mice were kindly provided by Dr. Juan Hidalgo, Universitat Autònoma de Barcelona). Wild type controls were either littermates that contained flanking loxP sites without the HSA-MCM construct or sex- and age-matched C57BL/6 J mice (The Jackson Laboratory Cat # 000664). Mice of both sexes were used in the studies and were allowed to mature to 8 weeks before being given intraperitoneal (IP) injections of tamoxifen **(**75 mg/kg/day for 5 days, Millipore Sigma, St. Louis, MO) in corn oil to induce gene recombination. Tamoxifen was allowed to washout for 3 weeks before subsequent experimental procedures were conducted.

To confirm recombination, mouse gastrocnemius muscle was lysed, and DNA was extracted using the Monarch Genomic DNA Purification Kit (New England Biolabs, Ipswich, MA). Samples underwent PCR using iTaq Universal SYBR Green Supermix (Bio-Rad, Hercules, CA) with the following primers: *Tlr4* Fwd—TCCTTGTTGCCCTTCAGTCAC, *Tlr4* Rev—CCCCTGGAAAGGAAGGTGTC; *Il6* Fwd—CCCACCAAGAACGATAGTCA, *Il6* Rev—ATGCCCAGCCTAATCTAGGT; *Ccl2* Fwd –TCT​ACA​CAG​CCC​CTC​CAT​GT, *Ccl2* Rev—AAGAGTGGGCCATTCACTCTC (Integrated DNA Technologies, Coralville, IA) using a C1000 thermocycler (Bio-Rad, Hercules, CA). PCR conditions were as follows: 1) 94°C 2:00, 2) 94°C 0:30, 3) 65°C 0:30, 4) 68°C 0:30, 5) repeat 2–4 10x, 6) 94°C 0:30, 7) 60°C 0:30, 8) 72°C 0:30, 9) repeat 6–8 28x, 10) 4°C. PCR products were subjected to ethidium bromide gel electrophoresis (1% agarose) and visualized using a GE Amersham 600 imager (GE Healthcare, Chicago IL).

### Acute Endotoxemia

To model acute endotoxemia, we administered 1.5 μg (1.0 μg per ml of blood volume, calculated to be approximately 1.5 ml per mouse (NC3RS, 2022)) of ultrapure O55:B5 lipopolysaccharide (Invivogen, San Diego, CA) in saline intravenously to mice via the retroorbital sinus. This LPS concentration was derived by determining in previous studies the leakage of LPS into the bloodstream subsequent to IT administration of 3 μg LPS/g in our mouse model of acute lung injury (Bivona et al., 2021). Validation of the IV LPS model was first performed in wild type mice that were administered IV saline vehicle or IV LPS followed by gastrocnemius muscle gene expression quantitation 3 h later, at which time mice were euthanized by an IP injection of 150 mg/kg pentobarbital (Euthasol, Midwest Veterinary Supply, Lakeville, MN). In subsequent experiments, wild type and gene-recombined mice were administered IV LPS and euthanized at 3 h by an IP injection of 150 mg/kg pentobarbital. Blood was collected *via* cardiac puncture, allowed to clot at room temperature, centrifuged at 21,300 x *g*, and serum was saved in tubes. The tubes containing serum and dissected gastrocnemius muscle were snap frozen in liquid nitrogen.

### Gene Expression Analysis

Gastrocnemius muscles were ground in liquid nitrogen using a mortar and pestle, and RNA was isolated using Trizol and chloroform (Thermo Fisher Scientific, Waltham, MA). Following purification, RNA was treated with DNAse I (Invitrogen, Waltham, MA) and quantified using a Nanodrop Spectrophotometer (Thermo Fisher Scientific). 100 ng of RNA was converted to cDNA using qScript cDNA Supermix (Quantabio, Beverly, MA) and gene expression was measured using iTaq Universal SYBR Green Supermix on a CFX96 Touch quantitative thermocycler (Bio-Rad). Gene expression relative to wild type (WT) controls was calculated using the 2^−ΔΔCt^ and normalized to expression of the housekeeping gene, beta-actin. Primer sequences for RT-qPCR were as follows: *Gapdh* Fwd — ACG​ACC​CCT​TCA​TTG​ACC​TC, Rev—TTCACACCCATCACAAACAT; *Tlr4* Fwd — CTG​GCT​GGT​TTA​CAC​GTC​CA, Rev—GCAGAAACATTCGCCAAGCA; *Il6* Fwd — TCC​GGA​GAG​GAG​ACT​TCA​CA, Rev—TTCCACGATTTCCCAGAGAACA; *Ccl2* Fwd — GAC​CCC​AAG​AAG​GAA​TGG​GTC, Rev—TGCTTGAGGTGGTTGTGGAAA; *Cxcl1* Fwd — GCT​GGG​ATT​CAC​CTC​AAG​AA, Rev—TGGGGACACCTTTTAGCATC; *Tnf* Fwd — TCC​CAG​GTT​CTC​TTC​AAG​GGA, Rev—GGTGAGGAGCACGTAGTCGG. PCR conditions were as follows: 1) 95°C 2:00, 2) 95°C 0:30, 3) 58°C 0:30, 4) 72°C 0:45, 5) repeat 2–4 39x, 6) 4°C.

### Serum Protein Quantification

Luminex based magnetic bead assays were used for protein quantitation of G-CSF, GM-CSF, IFN-γ, IL-1α, IL-1β, IL-5, IL-6, IL-9, IL-10, IL-12 (p40), IL-12 (p70), IL-13, IL-15, IL-17, IP-10/CXCL10, KC, LIF, LIX/CXCL5, MCP-1/CCL2, M-CSF, MIG/CXCL9, MIP-1α/CCL3, MIP-1β/CCL4, MIP-2/CXCL1, RANTES/CCL5, TNF-α, VEGF, and Eotaxin/CCL11 (Millipore Sigma) according to manufacturer’s instructions. All assays were performed in duplicate according to manufacturer’s instructions. Briefly, 25μL of serum diluted 1:5 or 1:50 in assay buffer, standard, or assay buffer (background) was added to each well of a black-walled 96-well microtiter plate. 25 μL of assay buffer plus 25 μL of conjugated beads were added to each well and the plates were covered, shaken vigorously for 1 min on an IKA (Wilmington, NC) MTS 2/4 digital microtiter plate shaker and then moderately shaken overnight at 4°C. After washing using a Bio-Rad (Hercules, CA) Bio-Plex Pro II wash station, 25 μL of biotinylated detection antibodies were added to the appropriate wells for 2 h followed by addition of 25 μL of streptavidin-PE to all wells for 30 min. The wells were washed and the beads were resuspended in 125 μL sheath fluid. Data from 50 beads per cytokine in each sample were acquired using the Bio-Rad Bio-Plex 200 suspension array system and Bio-Plex Manager 6.0 software. Fluorescence intensity of the background was subtracted from the values for each sample, standard, or control for each specific bead. Standard curves were generated from 5-fold dilutions of standards, which were analyzed using 5-place logistic regression from standards within 70%–130% of the expected values. Values from the 1:5 or 1:50 dilutions are presented based on analyte abundance in serum.

### Data Presentation and Statistical Analysis

Graphing and statistical analyses were performed on GraphPad Prism 9 (GraphPad Software, San Diego, CA). Data are presented as means ± SEM. For comparisons between two groups (e.g., saline versus LPS), Mann-Whitney t-tests were performed. For comparisons between multiple groups in which standard deviations were not equal, groups were compared using Brown-Forsythe and Welch ANOVA tests with Dunnett’s T3 multiple comparisons against wild type. When standard deviations were equal, ordinary one-way ANOVAs with Dunnett’s multiple comparisons were performed.

## Results

### Recombination of Floxed Genes in Myofibers

**FIGURE 1:**
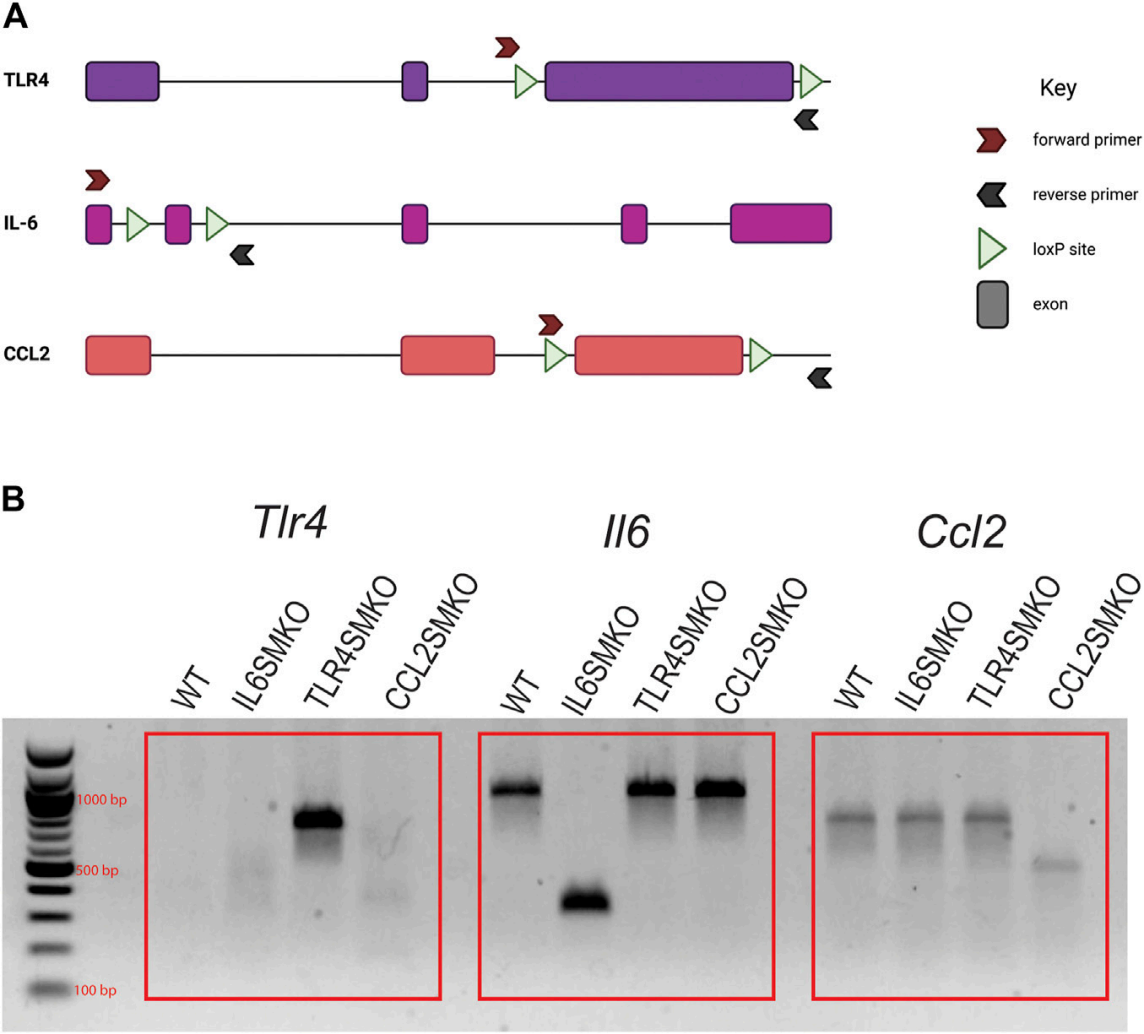
Confirmation of DNA recombination in skeletal muscle of targeted mice. Human skeletal actin MerCreMer mice were crossed with mice containing either floxed *Tlr4*, *Il6*, or *Ccl2* and given intraperitoneal injections of tamoxifen (75 mg/kg) for 5 consecutive days. **(A)** Simplified schematic of TLR4, IL-6, and CCL2 genomic constructs and PCR primer annealing sites. **(B)** PCR products from gastrocnemius muscle to confirm Cre-mediated gene recombination. Due to the size of exon 3 of TLR4, PCR amplification only occurs in recombined tissues. Data are representative of all mice tested (*n* = 11–39/group). Figure 1A was created using Biorender.com.

DNA was extracted from the gastrocnemius muscle of mice that underwent gene recombination and IV LPS injections. A brief schematic for floxed constructs and the PCR confirmation is shown in Figure 1A. Gene products, as visualized on agarose gels, are depicted in Figure 1B. The tissue specificity of the HSA-MCM mouse has been repeatedly shown to be localized to fully developed myofibers (McCarthy et al., 2012; Laitano et al., 2021a; Laitano et al., 2021b). Our results show abundant skeletal muscle recombination of *Tlr4*, *Il6*, or *Ccl2* specifically in the gene-targeted mice.

### Myofibers Constitute a Significant Proportion of LPS-Induced Cytokine-Producing Cells in LPS-Stimulated Skeletal Muscle

**FIGURE 2:**
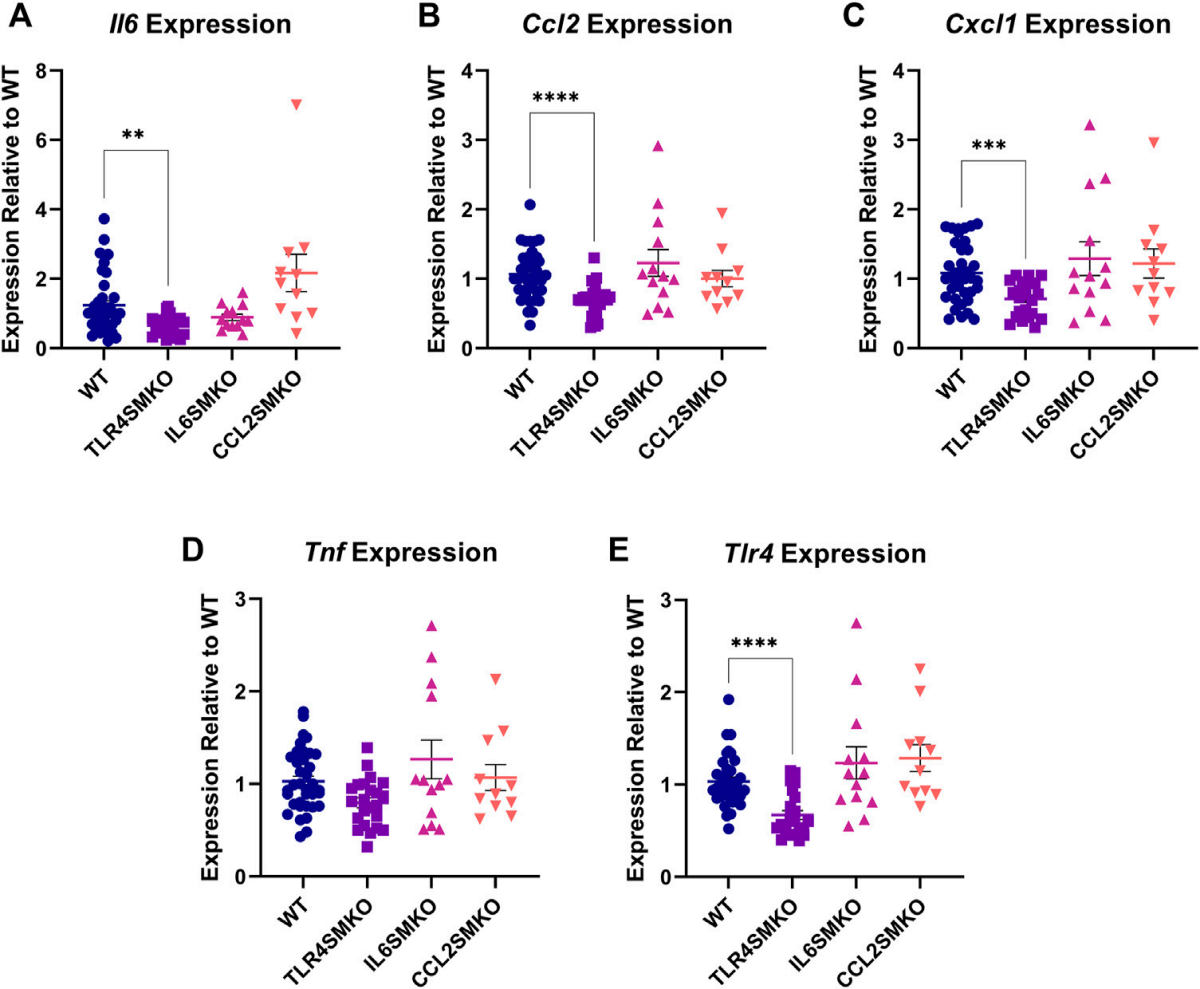
Skeletal muscle cytokine expression 3 h after IV LPS. Gastrocnemius muscle *Il6* **(A)**, *Ccl2* **(B)**, *Cxcl1* **(C)**, and *Tnf* **(D)**, as well as *Tlr4* **(E)**, from myocyte specific conditional knockouts for TLR4 (TLR4SMKO, *n* = 23), IL-6 (IL6SMKO, *n* = 13), CCL2 (CCL2SMKO, *n* = 11), or littermate controls (WT, *n* = 39). Values were analyzed using the 2^−ΔΔCt^ method relative to WT and compared using a one-way ANOVA with Dunnett’s multiple comparison test. *p* < 0.01 **, *p* < 0.001 ***.

Our earlier *in vitro* work reported the capacity of C2C12 myotubes to respond directly to LPS and to subsequently express and produce several pro-inflammatory cytokines (Bivona et al., 2021). To test whether these findings extend to the *in vivo* setting, we first established that, relative to saline administration, IV LPS administration induces expression in the gastrocnemius muscle of pro-inflammatory cytokines at 3 h (*Il6* = 128.19-fold (*p* < 0.0001); *Ccl2* = 6.06-fold (*p* < 0.0001); *Cxcl1* = 101.52-fold (*p* < 0.0001); *Tnf* = 2.24 (*p* = 0.0053), which is the time of maximal LPS-induced gene expression in skeletal muscle from earlier studies (Bivona et al., 2021). Next, gastrocnemius muscles from myofiber-specific *Tlr4*-, *Il6-*, or *Ccl2*-deleted mice were collected and analyzed for *Il6, Ccl2, Cxcl1, Tnf,* and *Tlr4* expression 3 h after IV LPS administration (Figure 2). TLR4SMKO mice showed a significant 33.25% decrease in the expression of *Tlr4*, accompanied by significant decreases in *Il6*, *Ccl2*, and *Cxcl1*. *Tnf* expression was unchanged in the muscle of knockout mice. Intriguingly, there were no significant decreases in *Il6* or *Ccl2* mRNA in IL6SMKO or CCL2SMKO mice.

### Myofibers Contribute to Systemic Cytokine Production

**FIGURE 3:**
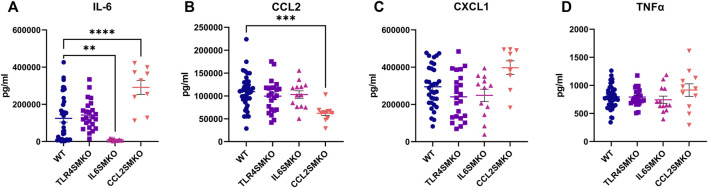
Skeletal muscle cytokine secretion influences systemic cytokine concentrations. Serum collected from mice containing myocyte specific conditional knockouts of TLR4 (TLR4SMKO, *n* = 23), IL-6 (IL6SMKO, *n* = 13), CCL2 (CCL2SMKO, *n* = 11), or littermate controls (WT, *n* = 39) was measured for concentrations of IL-6 **(A)**, CCL2 **(B)**, CXCL1 **(C)**, and TNFα **(D)** 3 h after IV LPS. Data were compared using one-way ANOVA with Dunnett’s multiple comparison test. *p* < 0.05 *, *p* < 0.01 **, *p* < 0.001 ***.

To determine the impact of myofiber-specific deletion of *Tlr4*, *Il6*, or *Ccl2* on systemic inflammatory tone, IL-6, CCL2, CXCL1, and TNFα were measured in serum collected 3 h following IV LPS challenge. Whereas TLR4SMKO mice showed no changes among these cytokines of interest compared to WT mice (Figure 3), IL-12 p40, IL-12 p70, and MIG/CXCL9 were significantly increased in TLR4SMKO mice (Supplementary Figure S1). In contrast, circulating IL-6 concentrations significantly decreased by 93% in IL6SMKO mice (Figure 3). IL-13 was also decreased in IL6SMKO animals (Supplementary Figure S1). Finally, CCL2 in serum from CCL2SMKO was reduced by 57% compared to WT mice (Figure 3). CCL2SMKO animals showed significant increases in serum IL-6 (Figure 3), as well as IL-1α, IL-1β, IL-6, IL-13, IL-15, IL-17, Eotaxin/CCL11, G-CSF, and LIF (Supplementary Figure S1). The knockout strains showed no differences in serum TNFα concentrations compared to WT mice (Figure 3). Assessment of serum cytokine concentrations in male and female mice used in the aforementioned studies revealed no significant sex-dependent differences in wild type, TLR4SMKO, IL6SMKO, or CCL2SMKO mice (Supplementary Figure S2).

## Discussion

For these studies, we implemented a low dose endotoxemia model to determine the extent to which myofibers contribute to the systemic inflammatory milieu with skeletal muscle specific knockdowns of a receptor (TLR4SMKO) and effectors (IL6SMKO and CCL2SMKO) involved in critical illness. We found that deletion of TLR4 from myofibers resulted in few changes in systemic cytokine concentrations during early endotoxemia. This observation agrees with our previous work that showed the requirement of macrophage derived TNFα to synergistically increase cytokine secretion from myofibers. Modest decreases in whole muscle *Tlr4* expression may be due to the influence of leukocyte *Tlr4* increases following IV LPS (Härter et al., 2004; Wan et al., 2016). Muscle resident leukocytes retain functional TLR4 in these knockout models, meaning the potential for myofiber-leukocyte crosstalk and synergy remains. The lack of circulating cytokine decreases contrasted gene expression in the gastrocnemius muscle of TLR4SMKO, in which *Il6, Ccl2, and Cxcl1* were reduced. Furthermore, IL6SMKO and CCL2SMKO groups had markedly different results. IL6SMKO mice had significantly reduced serum IL-6, indicating that skeletal muscle derived IL-6 contributes substantially to systemic concentrations of this cytokine. CCL2SMKO mice also had significant reductions in serum CCL2, but this decrease came with increases in serum IL-6, IL-1α, IL-1β, IL-13, IL-15, IL-17, Eotaxin/CCL11, G-CSF, and leukemia inhibitory factor (LIF). The contrast in gene expression and protein concentrations between the receptor and effector knockout models may be due to the ability of muscle to produce and secrete cytokines at a rate that is higher than other cell types. This is supported by our data as we observed the decreases in systemic IL-6 and CCL2, but absence of decreases in whole muscle gene expression of these cytokines, despite successful Cre-mediated gene deletion in myofibers. However, we cannot discount the contribution of other organ systems not investigated in this study.

Our model of acute endotoxemia mimics sepsis-like cytokine pathology and was derived from a model of IT LPS administration (Bivona et al., 2021). Lung leakage following injury allowed LPS to enter circulation and was measured systemically shortly after administration of the injurious LPS dose (Bivona et al., 2021). Therefore, to decrease potential confounding effects of lung injury, we used these measured systemic LPS values after IT LPS for our IV injections in the present study. Since IV LPS is cleared by the liver with a half-life of only 2–4 min (Yao et al., 2016), the model we employed provides a rapid burst of primary cytokines that is not perpetuated by infections, such as those present in cecal slurry or cecal ligation methods (Saito et al., 2003). Thus, our IV LPS model enabled us to determine skeletal muscle’s contribution to local and systemic pro-inflammatory biomarker/mediator production from the first TLR4-induced signaling events.

While recent studies have explored the involvement of myofibers in sepsis, they differ from the current studies in both the nature of the septic stimulus and the genetic models used. Laitano and colleagues reported the involvement of myofibers in sepsis using two similar knockout models, but with a different mouse model of sepsis. Using the same skeletal muscle-specific, tamoxifen-inducible deletion strategy, their group targeted MyD88 (Laitano et al., 2021b), one of the two adaptor proteins recruited to TLR4 upon its stimulation, and the cytokine IL-6 (Laitano et al., 2021a). Mice were injured with IP injections of cecal slurry and euthanized at 6 and 12 h. In the myofiber MyD88 knockout, there were varied decreases in pro-inflammatory responses, with decreases in TNFα and CCL2, but no changes in serum IL-6 or CXCL1 concentrations. In the myofiber IL-6 knockout, there were significant decreases in serum TNFα, IL-6, and CXCL1 concentrations. These decreases were accompanied by reductions in serum IL-10 and IL-4, which may account for increases in peritoneal neutrophils seen in both knockout models.

Whereas our experiments show differences at an early time point following IV LPS, the previous studies (Laitano et al., 2021a; Laitano et al., 2021b) report minimal to no differences until 6 h after injury. This disparity is likely due to the septic stimuli in the different models used in each study. The proliferation and migration of IP cecal bacteria may create a temporal delay from injection to muscle response, whereas IV injections of LPS are immediate and display more pronounced and rapid kinetics (Lewis et al., 2016; Seemann et al., 2017). In addition, sex differences were also reported in the studies of Laitano et al. Specifically, nearly all the decreases in cytokines were limited to female mice, with little to no change among male mice at both 6- and 12-h following injury (Laitano et al., 2021a; Laitano et al., 2021b). In contrast, there were minimal intragenotype sex dependent effects in our 3-h endotoxemia model reported herein. One limitation of our study was that mice were incorporated into experiments as they were born and matured over several months, so a possible contribution to the variability in our studies could be attributed to interexperiment or seasonal differences (Kiank et al., 2007).

There is vital importance to the onset of inflammation, particularly in stimulation of the compensatory anti-inflammatory response. If the response of anti-inflammatory cytokines such as IL-4 and IL-10 overcompensate for the initial wave of inflammation, it opens susceptibility for future infection or inability to fight the current one (Mathias et al., 2015). Since both pro-inflammatory cytokines and anti-inflammatory IL-10 are regulated by the transcription factor, NF-κB (Cao et al., 2006), a possible consequence of MyD88 or TLR4 removal from myofibers could be decreased compensatory, anti-inflammatory cytokines. However, cytokines are not the only mediators capable of resolving inflammatory events. Lipid based molecules such as resolvins and protectins have been shown to be vital in resolving muscle derived pro-inflammatory cytokines (Markworth et al., 2020). The enzymes responsible for synthesizing these anti-inflammatory lipid mediators have been found to be upregulated in muscle following LPS treatment, and future studies should investigate muscle’s role in resolving inflammation through this mechanism (Markworth et al., 2020).

While this and other studies measure decreases in systemic cytokine production in response to muscle-specific knockout of inflammatory sensors/signaling molecules/cytokines, they are unable to determine whether these effects are related solely to skeletal muscle release, or if other cell crosstalk intermediates are involved. Our earlier *in vitro* studies revealed the amplifying effects of macrophage derived TNF on LPS-induced pro-inflammatory cytokine production from co-cultured C2C12 myotubes (Bivona et al., 2021). The unchanged gene expression of gastrocnemius *Tnf* and systemic concentrations of TNFα suggest that this mechanism is still in play in our knockout models. This may partially explain the lack of systemic cytokine changes in the TLR4SMKO mice. While this was not directly investigated in this study, it is likely that a TNF-linked mechanism and additional cell-cell communication axes are at play in muscle *in vivo*. The contribution of resident leukocytes is evidenced by the insignificant decrease in muscle tissue *Ccl2* gene expression in the CCL2SMKO mice. We speculate that the absence of *Ccl2* decreases could be due to the increased expression of leukocyte derived transcripts during the LPS-induced inflammatory response. However, the systemic decrease in excreted CCL2 protein implies that myofibers may translate the cytokine to a substantial extent relative to the leukocyte population. Amongst the myriad of cell types that constitute skeletal muscle, myofibers appear capable of responding directly to pro-inflammatory stimuli and producing pro-inflammatory cytokines that contribute to systemic concentrations of these biomarkers. Further confirmation and identification of additional biomarkers could be accomplished using the recently-described secretome mouse, which is able to identify the cell type-specific origins of proteins (Liu et al., 2021), or by using a multi-omics approach and modeling genome-wide changes in muscle gene expression to changes in serum proteins. Regardless of whether other cell types contribute to the differences we observed using these knockout mice, or if the changes in systemic cytokines are directly due to myofiber deletions, the contribution of myofibers to modulation of both the local and systemic inflammatory tone appears substantial.

## Data Availability

The original contributions presented in the study are included in the article/Supplementary Materials, further inquiries can be directed to the corresponding author.
