# Supplementary material for: Skeletal Muscle Myofibers Directly Contribute to LPS-Induced Systemic Inflammatory Tone
Source: Front Pharmacol. 2022 Jun 23;13:917917. doi: 10.3389/fphar.2022.917917 (PMC9260049; doi:10.3389/fphar.2022.917917)
Supplement: Supplementary file 1 [file DataSheet1.DOCX]

Supplementary Material

## Supplementary Figures


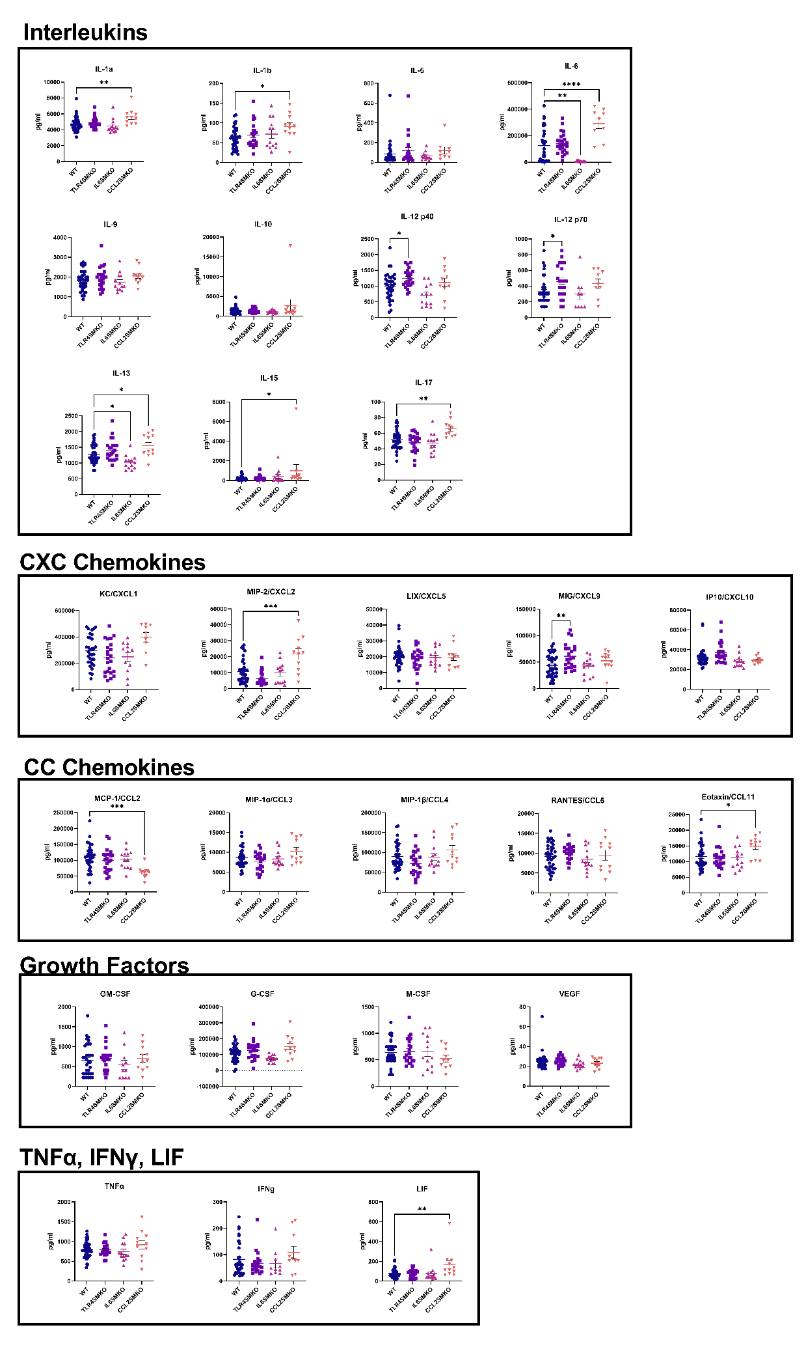


**Figure S1 Additional serum cytokines measured in the multiplex assay.** Serum collected from mice containing skeletal muscle specific conditional knockouts of TLR4 (TLR4SMKO, n = 23), IL-6 (IL6SMKO, n = 13), CCL2 (CCL2SMKO, n = 11), or littermate controls (WT, n = 39) was measured for concentrations of the indicated cytokines 3 hours after IV LPS. Data were compared using a one-way ANOVA with Dunnett’s multiple comparison test. p<0.05 *, p<0.01 **, p<0.001 ***, p< 0.0001 ****.


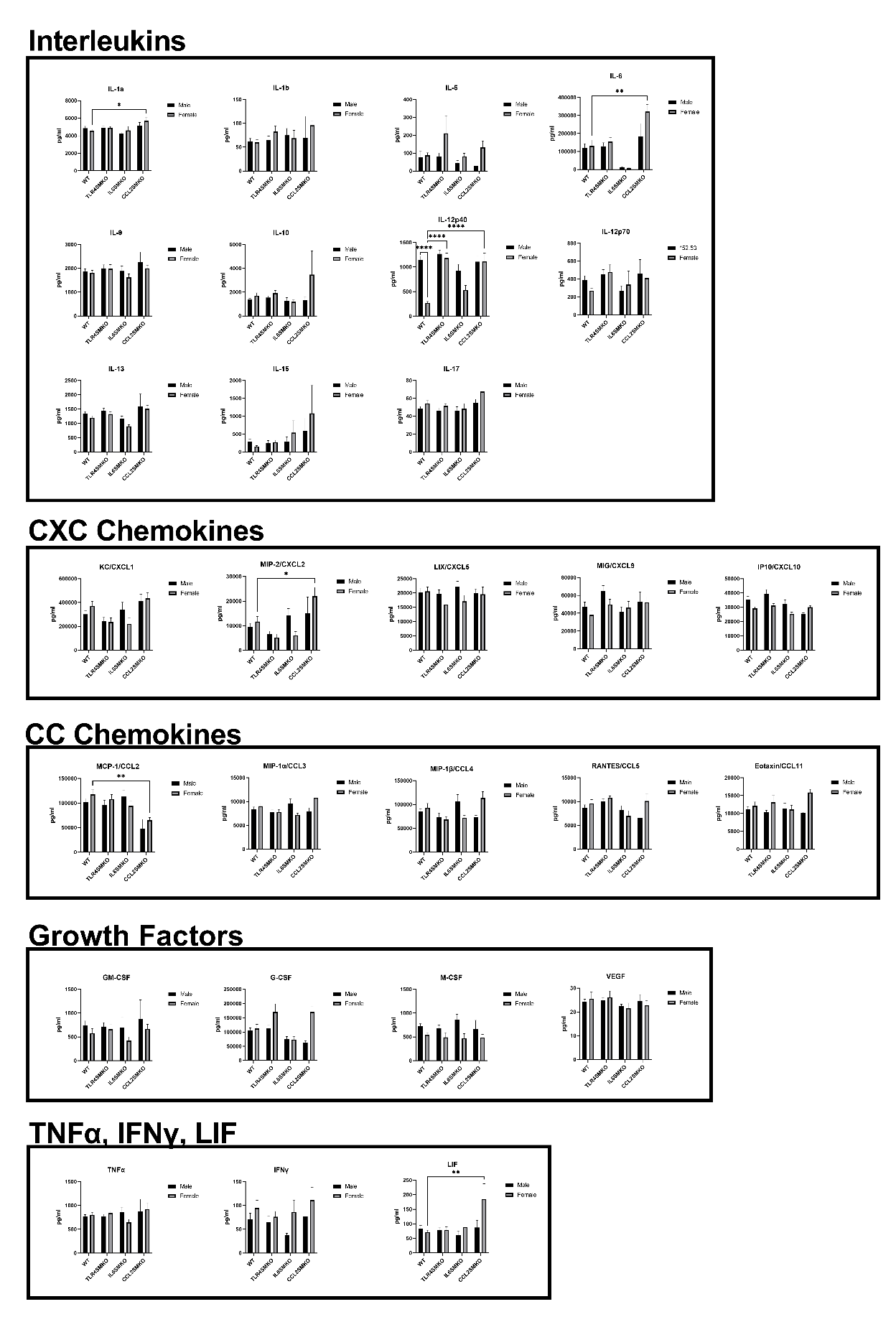


**Figure S2** **Evaluating the effect of sex on systemic cytokine levels.** Data from Figure 3 has been segregated by sex and evaluated using a two-way ANOVA with Sidak multiple comparisons p<0.05 *, p<0.01 **.
